# Management of a severely atrophic maxilla using concentrated platelet-rich fibrin block. A case report

**DOI:** 10.1093/jscr/rjae165

**Published:** 2024-03-17

**Authors:** Raghad N Saleh, Celine Ashhab, Meral Voltaire Kharoufeh, Cezar Edward Lahham

**Affiliations:** Oral and Maxillofacial Surgery Department, University College London, London EC4N 1SA, UK; Graduate Studies, Arab American University, Ramallah 00970, Palestine; Periodontics Department, Al-Quds University, Jerusalem 00970, Palestine; Periodontics Department, Arab American University, Bethlehem 00970, Palestine

**Keywords:** concentrated platelet-rich fibrin, bone allograft, horizontal bone augmentation, dental implant, maxillary atrophy

## Abstract

Tooth loss often leads to significant alveolar bone resorption, presenting a challenge for dental implant placement. This case report presents the effectiveness of concentrated platelet-rich fibrin (C-PRF) in combination with bone allograft for horizontal bone augmentation in a severely atrophic maxilla. A 33-year-old female patient with extensive bone loss in the upper anterior maxilla was treated in two stages. The initial stage involved horizontal bone augmentation using a mixture of C-PRF and bone allograft. This was followed, 5 months later, by dental implant placement. The preparation of C-PRF, surgical procedure, and postoperative care are thoroughly described. Post-treatment Cone Beam Computed Tomography showed an increase in alveolar bone thickness from 2.4–3.4 mm pre-operatively to 6.3–7.3 mm, demonstrating the procedure’s effectiveness in achieving adequate bone volume for implant placement. The use of C-PRF with allograft in horizontal bone augmentation shows promise in enhancing bone regeneration, especially in a severely atrophic maxilla.

## Introduction

Teeth loss typically causes physiological bone resorption, leading to reduced vertical and horizontal dimensions. Studies show a vertical bone loss of ~1.24 mm in the 6 months following tooth extraction and a horizontal bone loss of 3.8 mm [1]. Over a longer term, a study by Ashman reported a 40–60% reduction in the height and width of the alveolar bone within the first 2–3 years [2]. Insufficient bone volume can limit implant placement, requiring regenerative techniques to boost bone volume and guarantee implant success in the long term. The maintenance of alveolar bone volume is essential for implant success and long-term stability. There is evidence that bone preservation at the time of tooth extraction can minimize bone loss and contribute to better implant outcomes [3]. However, if there has been considerable bone resorption, it may be necessary to undertake horizontal bone augmentation to restore sufficient bone width for implant insertion.

Various regenerative techniques using advanced biomaterials have been introduced in the past to promote bone regeneration. These techniques include guided tissue and bone regeneration with barrier membranes, as well as a range of bone grafting materials from human, animal, and synthetic sources. In addition, bone morphogenetic proteins and enamel matrix derivatives have been combined to promote tissue healing and stimulate bone formation [4]. A commonly accepted method for promoting bone regeneration involves incorporating bone grafts with collagen membranes [5].

In recent years, platelet-rich fibrin (PRF) has emerged as a promising autologous regenerative material in dentistry. PRF is prepared by a simple centrifugation process without the use of any anticoagulants. The fibrin matrix in PRF is enriched with platelets, leukocytes, growth factors, and cytokines [6]. The distinctive composition of these bioactive components makes PRF a promising candidate for enhancing tissue regeneration. Fibrin networks are formed during coagulation and synergize with cytokines released by platelets to create a biocompatible matrix that is proficient in retaining tissue growth factors [7]. Osteoblasts, endothelial cells, chondrocytes, and fibroblasts can be stimulated and differentiated by these factors, which can assist in the formation of bones and the healing of soft tissues [8, 9]. Recently, a novel formulation called concentrated platelet-rich fibrin (C-PRF) has emerged as an extension of the traditional PRF. By utilizing a modified centrifugation protocol, C-PRF is acquired with a liquid consistency and a high concentration of platelets and growth factors. This distinctive feature may enhance bone regeneration by making it easier to apply and more adaptable to irregular bone defects [10]. There has been recent research on enhancing the effectiveness of PRF in horizontal bone augmentation procedures. While conventional (PRF) has shown promising results, researchers are exploring alternative methods to further enhance its effectiveness [11, 12].

This case report investigates the utilization of bone allografts in conjunction with concentrated platelet-rich fibrin (C-PRF block) for horizontal bone augmentation. This is in light of PRF’s evolving potential and the advent of C-PRF. This innovative approach aims to enhance the effectiveness of bone regenerative procedures and address bone deficiencies. Throughout this report, we will present a detailed clinical case, describing the treatment procedures and outcomes, as well as a thorough analysis of the results that were obtained.

## Case report

**Figure 1:**
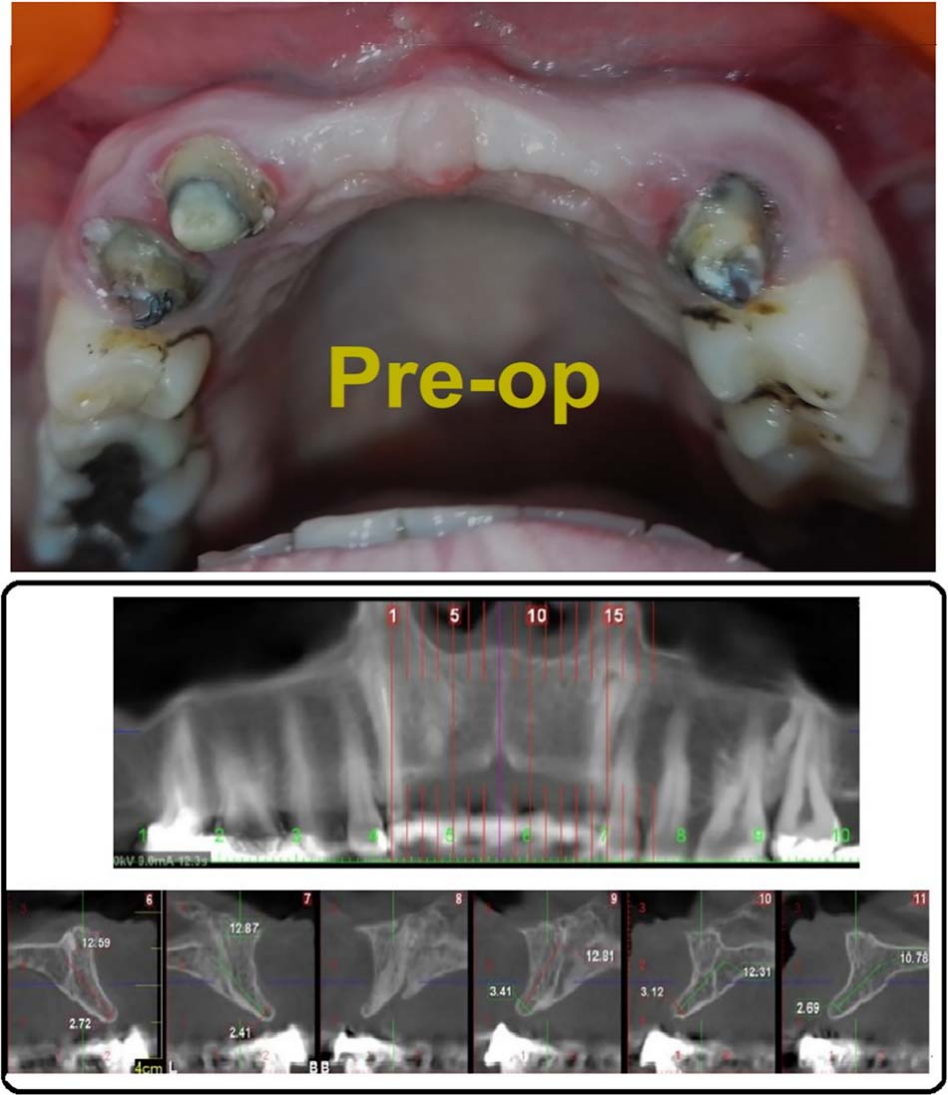
(A) Pre-operative photograph, (B) pre-operative CBCT.

A 33-year-old medically fit female patient came to the dental care center suffering from missing teeth in the upper anterior region (11, 21, 22). During clinical and radiographic examinations, severe bone resorption was observed. Alveolar bone thickness ranged from 2.4 to 3.4 mm (Class IV according to Tolstunov classification for alveolar ridge defects [13]), (Fig. 1A and B) gingival thickness was moderate (1 mm). After discussing all treatment options with the patient, the treatment was planned in two stages. The first stage, horizontal bone augmentation using bone allograft in combination with C-PRF. The second stage, implant surgery after 5 months from the first stage.

### C-PRF preparation

After obtaining an informed consent, 18 ml of venous blood was collected from the patient in plain white-cap tubes (9 ml, VACUETTE® tube). Then, blood was centrifuged at high g-force (700 g) for 8 min. A total of 0.5 ml of concentrated PRF was collected from the buffy coat from each tube.

### Operation

**Figure 2:**
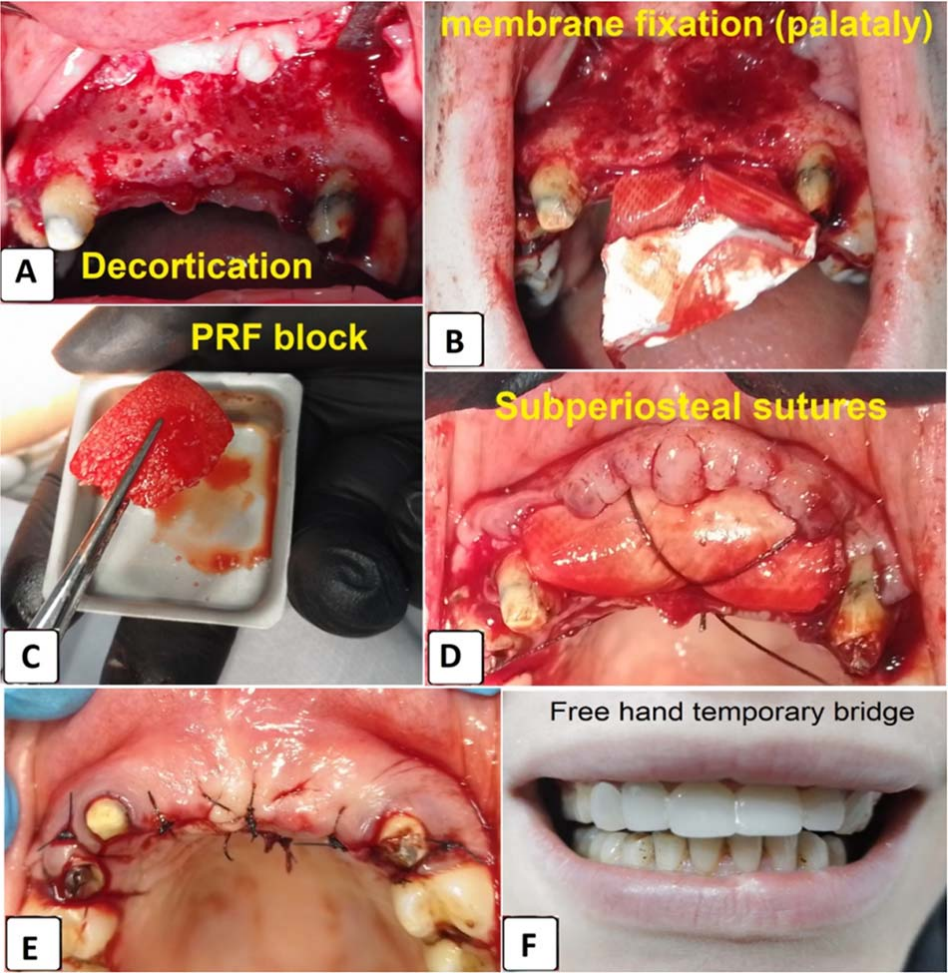
(A) Decortication, (B) collagen membrane fixation, (C) C-PRF Block, (D) collagen membrane fixation using resorbable sutures, (E) flap replacement and suturing, (F) temporary bridge placement.

A 2% lidocaine with 1:100000 epinephrine was given. A mid-crestal incision with two releasing incisions was done and a full-thickness flap was elevated. Then, decortication was performed using small diamond round bur (Fig. 2A). Following that, collagen membrane was fixated apically (Fig. 2B).

**Figure 3:**
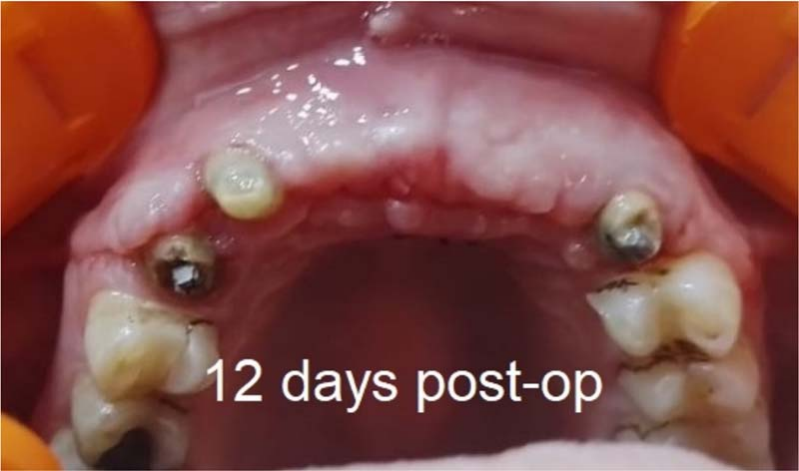
12 days postoperative.

A total of 1 ml C-PRF was mixed with 2.5 cc bone allograft (Dizg®). This mixture was left for 10 min for solidification and became C-PRF block (Fig. 2C). The graft was placed on the decorticated bone under the collagen membrane. The graft was covered with a collagen membrane and fixed palatally (Fig. 2D). Finally, the flap was replaced and sutured using 4–0 silk suture (Fig. 2E, F). The sutures were removed 12 days following surgery (Fig. 3).

**Figure 4:**
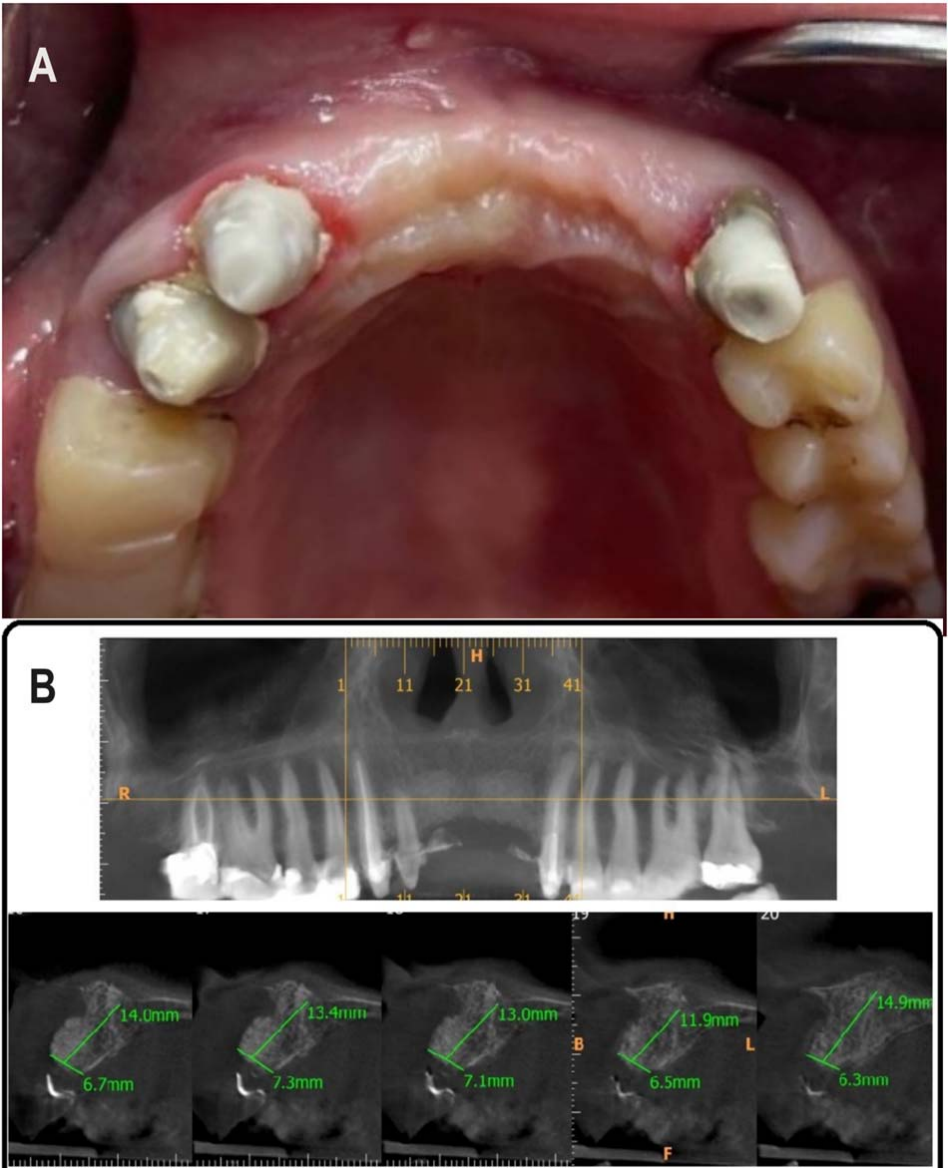
(A) 5 months postoperative photograph, (B) 5 months postoperative CBCT.

After 5 months, Cone Beam Computed Tomography (CBCT) was done to assess the thickness of the alveolar bone. Alveolar bone thickness was increased 4–5 mm from the baseline, and it ranged from 6.3 to 7.3 mm (Class II according to Tolstunov classification for alveolar ridge defects [13]), which was sufficient to place dental implants (Fig. 4A and B).

## Discussion

This study evaluated the clinical effectiveness of incorporating C-PRF into bone allografts to enhance bone gain in cases of severe maxillary atrophy. Our findings indicate a promising outcome, aligning with several studies that have demonstrated the efficacy of PRF in conjunction with bone allografts [14]. Specifically, our case showed an increase in bone thickness, which is consistent with reported bone gains of 2.7 ± 0.1 mm using similar techniques [15]. Bone graft with collagen is also commonly used for guided bone regeneration. In a systematic review, the amount of bone gain was 3.8 + 0.8 [16]. However, no previous clinical study evaluated the effectiveness of C-PRF block for ridge augmentation.

**Figure 5:**
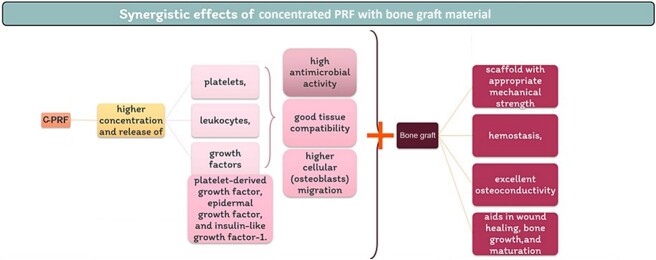
Synergistic effects of concentrated PRF with bone graft material.

Although PRF has many preparation protocols, there is evidence that C-PRF has the highest capacity to release growth factors for a long duration [17]. Moreover, combining C-PRF with bioactive materials, such as bone allograft, offers several advantages (Fig. 5). First, it serves as a scaffold material with appropriate mechanical strength, hydrophilicity, and excellent osteoconductivity. Additionally, it aids in wound healing, bone growth, maturation, material stabilization, and hemostasis. Lastly, this combination improves the handling properties of bioactive materials [18, 19].

However, our study is not without limitations. As a single case report, the findings need to be validated with larger sample sizes and controlled studies. Furthermore, the long-term stability and functionality of the augmented bone, beyond the immediate postoperative period, remain to be evaluated through follow-up studies.

Future research should focus on comparative studies that evaluate C-PRF with other regenerative materials, as well as long-term follow-up studies to assess the durability of the augmented bone. Additionally, investigations into the patient-specific factors that might influence the outcome of such procedures are necessary to tailor treatments to individual needs.

In clinical practice, the use of C-PRF in conjunction with bone allografts could revolutionize the approach to managing severe maxillary atrophy, especially in cases where traditional bone augmentation techniques might be insufficient. This approach could potentially reduce treatment times, enhance patient comfort, and improve overall treatment outcomes [6].

In conclusion, our study highlights the potential of C-PRF as an effective adjunct in bone grafting procedures, offering a promising direction for future research and clinical applications in dental implantology.

## Conclusions

This study demonstrates the potential of C-PRF combined with bone allograft in enhancing bone regeneration for cases of severe maxillary atrophy. The integration of C-PRF with bone allografts has shown promising results in improving the quantity and quality of bone gain, which is crucial for successful dental implantology. The use of C-PRF not only enhances the predictability of bone augmentation outcomes but also facilitates the surgical handling and manipulation of graft materials. The distinct advantages of C-PRF, such as its sustained release of growth factors and its synergistic effects with bone allografts, position it as a valuable tool in regenerative dental procedures. Its role as a scaffold material and its osteoconductive properties further underscore its potential in addressing complex dental cases involving significant bone loss.

In summary, the findings of this study suggest that C-PRF, in combination with bone allografts, offers a novel approach for effective bone regeneration in dental implantology. This technique holds promise for enhancing patient outcomes and expanding the range of treatment options in the field.
